# Effectiveness of Resective Surgery in *Complex Ameloblastoma* of the Jaws: A Retrospective Multicenter Observational Study

**DOI:** 10.3390/cancers14194608

**Published:** 2022-09-22

**Authors:** Davide Sozzi, Andrea Cassoni, Elena De Ponti, Mattia Moretti, Resi Pucci, Davide Spadoni, Gabriele Canzi, Giorgio Novelli, Valentino Valentini

**Affiliations:** 1 Maxillofacial Surgery Unit, ASST Monza—San Gerardo Hospital, 20900 Monza, Italy; 2 Department of Medicine and Surgery, School of Medicine and Surgery, University of Milano Bicocca, 20900 Monza, Italy; 3 Oncological and Reconstructive Maxillofacial Surgery Unit, Policlinico Umberto I of Rome, 00161 Rome, Italy; 4 Department of Oral Maxillofacial Sciences, Sapienza University of Rome, 00185 Rome, Italy; 5 Department of Medical Physics, ASST Monza—San Gerardo Hospital, University of Milano Bicocca, 20900 Monza, Italy; 6 Postgraduate School of Maxillofacial Surgery, University of Milan, 20122 Milan, Italy; 7 Maxillofacial Surgery Unit, ASST Santi Paolo e Carlo—Ospedale San Paolo, 20142 Milan, Italy; 8 Maxillofacial Surgery Unit, Emergency Department, ASST Grande Ospedale Metropolitano Niguarda, 20162 Milan, Italy

**Keywords:** ameloblastoma, odontogenic tumor, jaw tumor, resective surgery

## Abstract

**Simple Summary:**

Ameloblastomas are rare, benign, progressively growing epithelial odontogenic tumors with a high tendency to relapse. Although numerous studies concerning the treatment of these tumors have been published, there is still no unanimity. This study seeks to confirm whether radical surgery is more effective in avoiding relapses. We included 55 patients affected by *complex ameloblastoma*, characterized by recurrence, soft-tissue involvement, erosion of internal/external cortical walls with involvement of the inferior margin of the mandible, and/or invasion of the maxillary sinus or nasal cavity. All patients underwent wide surgical resection and were then followed for an average of 108 months, with a recurrence rate of 10.9%. Maxillary ameloblastoma has an increased risk of recurrence, especially in patients who underwent previous surgical treatments. Our results suggest that complete resection could prevent the onset of relapse. However, this treatment seemed less effective in preventing recurrences in the soft tissues or maxillary sinus.

**Abstract:**

Ameloblastoma is a rare, benign, odontogenic tumor of epithelial origin, characterized by locally aggressive, expansive growth. Treatment is controversial due to the risk of relapse. The aim of this multicenter retrospective study was to evaluate the effectiveness of complete resection in cases of *complex ameloblastoma*, which is considered at a higher risk of recurrence. Patients who met at least one of these criteria were included: recurrence, soft-tissue involvement, complete erosion of internal/external cortical walls with involvement of the inferior margin of the mandible, and invasion of the maxillary sinus or nasal cavity. Demographic data, tumor site, type of surgery, histological features, and follow-up information were collected for each patient. The cohort included 55 patients with a mean follow-up of 108 ± 66 months. A multivariate logistic model was used to evaluate variables independently associated with relapse. There were six soft-tissue or maxillary sinus relapses, with a recurrence rate of 10.9%. Most of them arose in patients previously treated. The statistical analysis identified the maxillary location as a fundamental relapse risk factor. En bloc resection with large surgical safety margins seemed to be effective in preventing the relapses. However, complete resection was less effective in preventing recurrences in the soft tissues or maxillary sinus.

## 1. Introduction

Ameloblastoma is a rare benign odontogenic tumor of epithelial origin, with an estimated annual incidence of about 0.92 cases per million [1], characterized by locally aggressive, expansive growth, and a high tendency for relapse. Malignant transformation represents 2% of the cases, with a not negligible ability to metastasize [2,3]. This type of tumor generally affects the mandible (80% of cases), with no predilection for males or females, and the median age of presentation is approximately 35 years [4,5]. Ameloblastoma is generally asymptomatic: it may be discovered incidentally during imaging studies performed for other reasons. As the size of the lesion increases, the earliest manifestations include a slow-growing, generally painless swelling, followed by changes in the surrounding dental elements, invasion of the soft tissues of the mouth with subsequent facial deformities, functional limitations, and, in some cases, paresthesia and alterations in sensitivity [6]. The treatments currently recognized in the scientific literature include wide surgical excision with large resection margins, which may or may not be associated with reconstruction, and conservative surgery [7,8,9]. Often the diagnosis of ameloblastoma occurs after conservative surgical treatment because a differential diagnosis from odontogenic cysts is difficult, these lesions are locally less aggressive, and, therefore, do not require a wide surgical resection. However, in the latter case, there is a high incidence of recurrence (60–80% of cases) [10]. More than 50% of relapses occur within five years after surgery, but it is advisable to consider a life-long follow-up [5] because some relapses have been described even more 25 years after the resective treatment. The treatment of recurrent ameloblastoma often requires a very extensive resection, which involves more bone segments than the primary resection. Soft tissues are also more often involved. Furthermore, relapses that extend to the intracranial level always have important resective and reconstructive limits. Both the resection and the reconstruction, if, and when, it is necessary to treat this type of relapse, become very complex [11]. However, mutational status analysis is enabling new therapeutic perspectives, especially in the case of mutations in the BRAF gene, which is the gene most frequently connected with metabolic alterations that give rise to the disease. Some authors do, indeed, affirm that the use of BRAF inhibitors could result in tumor regression, allowing for less invasive surgery with organ and bone preservation [12,13,14,15]. Recently, many case series have been published, but only a few articles investigate the outcomes of radical surgical treatments and subsequent reconstructive options in ameloblastomas that could be defined as *complex*—namely, requiring a resection on safety margins because of their extension and site or characterized by multiple relapses and, therefore, with a greater risk of further recurrence [9,10,16,17]. We classified as *complex ameloblastomas* the cases that met at least one of these characteristics: recurrence, involvement of the soft tissues of the mouth, erosion of internal/external cortical walls with involvement of the inferior margin of the mandible, and invasion of the maxillary sinus or nasal cavity. Consistent with this line of thought [10,18,19], this study aims to evaluate the effectiveness of complete resection in preventing relapses in a subgroup of ameloblastomas characterized by a greater risk of recurrence. Secondly, clinical and histopathological characteristics of the neoplasms included in our sample have been analyzed in order to identify possible risk factors for relapse.

## 2. Materials and Methods

A retrospective, multicenter, observational study was conducted on patients who underwent extensive surgical ablation treatment, following a diagnosis of *complex ameloblastoma* of the jaw, at the Operative Unit of Maxillofacial Surgery of the ASST Monza San Gerardo Hospital at the University of Milano Bicocca and at the Maxillofacial Surgery Unit of the Policlinico Umberto I at the Sapienza University of Rome, in the period between January 1997 and December 2019. This research study was conducted retrospectively from data obtained for clinical purposes. We consulted extensively with the members of our Institutional Review Board, who determined that our study did not need ethical approval. The study was conducted in accordance with the Declaration of Helsinki, and written informed consent was obtained from all subjects involved in the study.

In accordance with the characteristics of *complex ameloblastoma*, the following were included: (1) recurrence of ameloblastoma, (2) involvement of the soft tissues of the mouth, (3) erosion of internal/external cortical walls with involvement of the inferior margin of the mandible, and (4) invasion of the maxillary sinus or nasal cavity. Patients without a minimum follow-up of 12 months were not included in our study. We analyzed medical records, outpatient records, reports of surgical interventions, and radiological documentation of each patient. Demographic data, characteristics of the surgery (anatomical site affected, extent of bone resection, absence or presence of reconstruction, and type of reconstruction performed), morphological and histological conditions of the disease (histological type, size, and negativity or positivity of resection margins), and postoperative information (follow-up duration and possible relapses) were collected. Statistical analysis was conducted using absolute numbers and percentages in the case of categorical parameters, while continuous variables were summarized using mean value, standard deviation, median, and interquartile range.

The association between variables and outcomes, intended as the efficacy of the therapy in terms of relapse rate, was studied through Fisher’s exact test for dichotomous and categorical variables and Wilcoxon’s rank test for continuous variables. Stata software 9.0 (Stata Corporation, College Station, TX, USA) was used for performing statistical analysis (D.P.E.).

## 3. Results

A cohort of 55 patients was included in the study. The characteristics of the sample are summarized in Table 1: mean age was 47.2 ± 17.2 years (range: 16–85 years); 30 patients were male (54.5%) and 25 patients were female (45.5%).

Twenty-seven of the patients included in our sample (49.1%) came to our attention with a relapse, after having been treated in other centers. In all cases, it was possible to gather information on the therapeutic interventions they had previously undergone, as shown in Table 2. As already mentioned, after histological confirmation obtained by incisional biopsy, all patients underwent en bloc resection with large macroscopic margins on healthy tissue.

The sizes of the ameloblastomas are indicated in Table 3, together with the involvement of the resection margins, which were evaluated in 52 cases (94.6%), and the histological patterns [20].

As illustrated in Table 4, 44 patients (80.0%) underwent immediate reconstructive surgery (reconstructive plates, bone grafts, temporalis muscle flaps, and free flaps). Eleven patients (20.0%) did not undergo any reconstruction.

Information about the postoperative follow-up is available for all patients, with an average time of 108 ± 66 months (range: 12–244 months; median: 102 months; IQ range: 45–166 months). Table 5 highlights the main differences between the patients who developed relapses and those who remained disease-free. Analyzing the six cases of relapse in the cohort under study (10.9%) and the relationship between relapse and disease localization, we can state that in four cases (66.7%), the relapse arose in the context of the maxillary bone, and in the mandible in two cases (33.3%). In three of the six cases of recurrence (50%), the relapse arose in soft tissues, despite the wide surgical excision with safety margins previously performed. In the remaining three cases (50%), the relapse arose in the maxillary sinus, and the cranial base was also involved.

**Figure 1:**
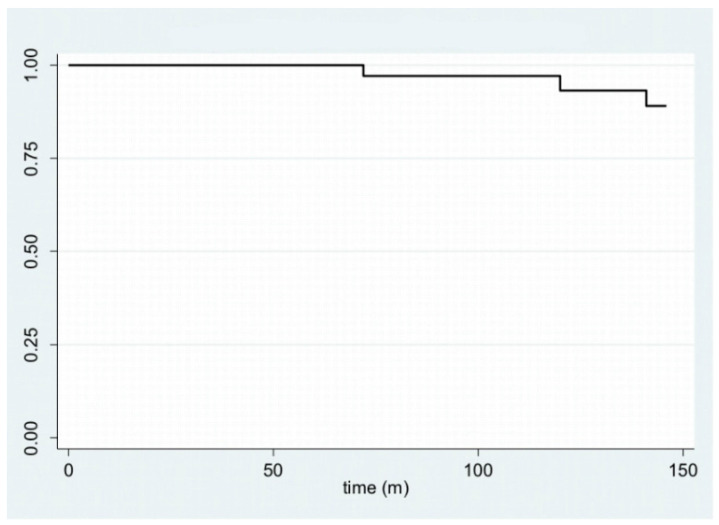
Kaplan–Meier plot for disease-free survival.

**Figure 2:**
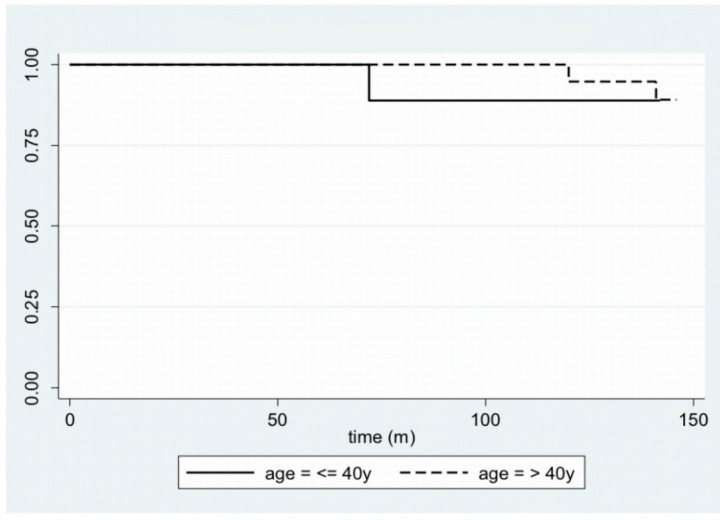
Kaplan–Meier plot for disease-free survival, stratifying the sample by age.

**Figure 3:**
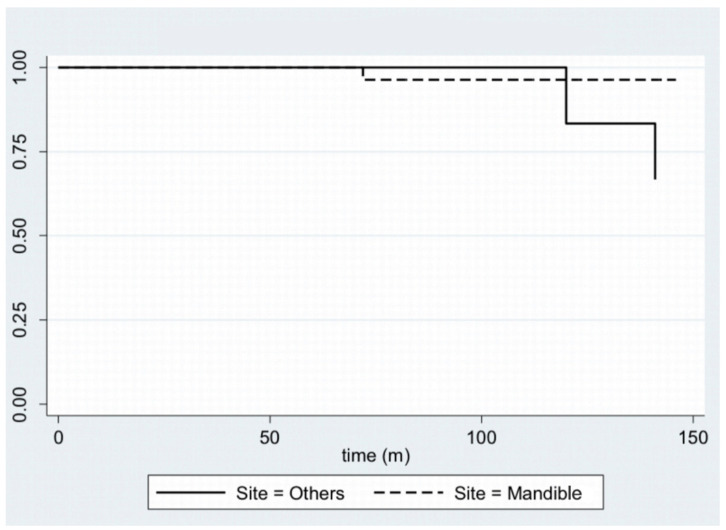
Kaplan–Meier plot for disease-free survival, stratifying the sample by site.

In Figure 1, disease-free survival is displayed with a Kaplan–Meier plot. Furthermore, by stratifying the sample by age—using 40 years as the cut-off age—and site, it is possible to plot the Kaplan–Meier curves (Figure 2 and Figure 3).

Finally, a multivariate logistic model was used to evaluate what variables may be independently associated with relapse: the characteristics of the patients (age and anamnesis), disease (site, size, and histological type), and surgical treatment. We discovered that the only variable independently associated with the onset of relapse is the anatomical site, the maxillary location in particular. Table 6 displays the odds ratios (OR)—relating to the variables described with a confidence interval of 95%—and the *p*-values for both the univariate and multivariate logistic analyses.

## 4. Discussion

Ameloblastoma is an odontogenic tumor and, unlike most benign tumors, has characteristics that often complicate the patient’s clinical status in the long term: a marked tendency to relapse (8–90%), the possibility of malignant degeneration (2%), and the ability to metastasize at a distance (<1%) [2,3,21,22]. Notably, ameloblastomas can cause recurrence after disease-free intervals of even 25 years [20]; indeed, a case report describes a relapse that arose 50 years after the first diagnosis [23]. For this reason, it is necessary to refer patients to long-lasting follow-ups [24]. According to many authors, the global recurrence rate of ameloblastoma is around 10% and increases to values between 55% and 90% in the case of conservative surgical treatment [10,25,26,27,28,29,30,31]. This behavior can be explained by the fact that the smallest amount of residual disease is sufficient to cause its recurrence. Therefore, extensive en bloc resection with appropriate reconstruction is the best course of action to lower recurrence rates. This claim is also confirmed by McClary et al. [32], who examined different studies and highlighted how radical treatment has a significantly lower relapse rate, equal to 11%, compared to conservative treatment, with a relapse in 65% of cases. The recent meta-analysis conducted by Hendra et al. [18] states that, in solid pattern tumors, radical treatment leads to a relapse rate of about 8%, whereas conservative treatment leads to relapse in 41% of cases. However, they do not describe whether the cases treated are primary or secondary. The aim of our study was to evaluate radical treatment in cases of *complex ameloblastoma*, providing criteria for identifying when radical treatment is absolutely necessary to reduce the risk of recurrence. We classified as *complex ameloblastoma* the cases that met at least one of these characteristics: recurrence, involvement of the soft tissues of the mouth, erosion of internal/external cortical walls with involvement of the inferior margin of the mandible, and invasion of the maxillary sinus or nasal cavity. According to Yang et al. [26], soft-tissue invasion and maxillary sinus involvement are risk factors for recurrence. Another fundamental factor in predicting the risk of recurrence is the tumor site; in fact, the contiguity of the maxilla with the pterygopalatine fossa, the infratemporal fossa, and the skull base makes this bone a more common site of recurrence [11]. Our data—four out of the six cases of relapse in our cohort (66.7%) arose in the context of the maxilla—together with the univariate and multivariate analyses we performed, confirm this concept. The management of the lesions in these districts always represents a challenge, especially in obtaining wide free resection margins and performing a satisfactory reconstruction [11]. The relapse rate that emerges from our study, equal to 10.9%, seems to agree with the data in the literature, despite our cohort having a higher risk of recurrence. Hong et al. point out that the histological pattern is also related to the risk of recurrence: the follicular histotype seems to have the highest relapse rate (26.3%) according to Armocida et al. [11], followed by the plexiform histotype (21.7%) [33]. Taking into consideration the six patients with relapse presented in our study, two of them presented a follicular histotype (33.3%), and one presented a plexiform histotype (16.7%); the remaining three patients (50.0%) presented other histotypes. Therefore, although not statistically significant, the data presented here would seem to be in line with the scientific literature previously cited. Yang et al. also demonstrated the association of tumor size with recurrence rate: the greater the size and the invasion of adjacent structures, the more likely the recurrence [10]. In this case, the sample in our study, probably because of the reduced number of relapses involved, would seem to go against this statement; in fact, there is no difference in the size of ameloblastomas with a tendency to relapse (3.2 cm ± 1.8 cm) and the size of neoplasms that do not recur (3.5 cm ± 1.6 cm). Finally, our data suggest the absence of a correlation between age and relapse; the only trend that can be inferred concerns an earlier relapse in young patients, as shown in Figure 2. However, we have found no difference between young and elderly patients in the long term.

Ameloblastomas have a low incidence [1], which makes it difficult to obtain statistically significant results. The benignity of these lesions often suggests a less aggressive treatment, but the course of treatment should be carefully planned in consideration of the high rate of relapse. It is not currently possible to consider any approach as the most appropriate for this reason. However, en bloc tumor resection with wide free surgical margins, followed by simultaneous or deferred reconstruction, is considered by experts the gold standard of care [10,18,26,27,34,35]. The type of reconstruction varies according to the location and extent of the defect. The reconstructive techniques we used in our cohort ranged from reconstructive plates to locoregional flaps (such as the temporal muscle flap) to the harvest of free revascularized flaps. Immediate reconstruction with microsurgical flaps guarantees the best aesthetic and functional result. The bone flaps, such as the fibula free flap and the iliac crest flap, allow the restoration of bone. As affirmed by many authors, fibula-free flaps guarantee a satisfying result in reconstruction, with a higher success rate, greater stability, and greater reliability for dental rehabilitation [36,37,38,39,40,41,42]. Furthermore, the use of revascularized bone flaps allows implant-prosthetic rehabilitation, which is correlated with a significant improvement in the quality of life of the patients [42].

In any case, the professionals must consider both the characteristics of the pathology, such as size, site, and local invasiveness, and the characteristics of the patient, such as age and comorbidities, when choosing the best procedure to perform.

Our study supports the line of thought that radical surgery is more effective than the conservative approach in preventing the onset of relapse. However, in multi-recurrent cases, not even wide surgical excision is fully adequate, especially when it is used to treat relapses in the soft tissues or paranasal sinus: five out of the six cases (83.3%) of relapse involved in our study were multi-recurrent ameloblastomas, characterized by a long history of treatments prior to being referred to our departments.

This study has some limitations: some data are not statistically significant because of the small number of patients enrolled, as it often happens in rare tumors. Also, as this is a retrospective study, many cases were not included because the information available to us was incomplete. Moreover, molecular and genetic analyses were not processed due to a lack of clinical use. Finally, the quality of life of patients after surgery was not assessed by questionnaires evaluating the impact of surgical treatments.

## 5. Conclusions

Ameloblastoma is a rare disease that influences the lives of the affected patients because of its innate tendency to recur. Our study allows us to conclude that a radical treatment guarantees a lower possibility of recurrence, even in complex cases with a higher risk of relapse, classified as *complex ameloblastoma* the cases that met at least one of these characteristics: recurrence, involvement of the soft tissues of the mouth, erosion of internal/external cortical walls with involvement of the inferior margin of the mandible, and invasion of the maxillary sinus or nasal cavity. Our multivariate logistic model confirms that recurrences are certainly favored by a maxillary location and often arise in the soft tissues or maxillary sinus. In such cases, not even a wide surgical resection with safety margins is fully effective in erasing the risk of relapse in the long term. However, given the limitations of our research, collaboration and sharing of homogeneous data from several departments involved in collective studies could be fundamental to carrying out detailed and unequivocal assessments, performing multivariate statistical analyses, and examining in depth the impact of this pathology and its treatments on the patient’s life.

## Figures and Tables

**Table 1:** Cohort characteristics.

	Categories	Data
Age	≤40	17 (30.9%)
	>40	38 (69.1%)
Gender	Male	30 (54.5%)
	Female	25 (45.5%)
Site	Mandible	44 (80.0%)
	Maxilla and Infratemporal fossa	10 (18.2%)
	Fronto-ethmoidal region	1 (1.8%)

**Table 2:** Previous treatments in patients with ameloblastoma relapse.

Variables	Categories	Data
History of ameloblastoma	Yes	27 (49.1%)
	No	28 (50.9%)
Previous treatments	Conservative treatment	18 (66.7%)
	Radical surgery	5 (18.5%)
	Conservative treatment + radical surgery	4 (14.8%)

**Table 3:** Size, resection margins, and histological patterns of the ameloblastomas.

Variables	Categories	Data
Size	Mean ± SD (range)	3.4 ± 1.6 cm (0.8–7.5 cm)
	Median (IQR)	3.0 cm (2.5–4.0 cm)
Resection margins	Negative	45 (81.8%)
	Positive	7 (12.7%)
	Not known	3 (5.5%)
Histological patterns	Follicular type	30 (54.6%)
	Plexiform type	7 (12.7%)
	Other	18 (32.7%)

**Table 4:** Type of reconstruction.

Variables	Categories	Data
**Type of reconstruction**	Free flap	25 (56.8%)
	Bone graft	11 (25.0%)
	Temporalis muscle flap	5 (11.4%)
	Reconstruction plate	3 (6.8%)

**Table 5:** The main differences between patients who developed relapses and patients who remained disease-free.

Variables	Categories	Relapse	No Relapse	*p*-Value
Age	≤40	1 (16.7%)	16 (32.7%)	0.39
	>40	5 (83.3%)	33 (67.3%)	
Sex	M	3 (50.0%)	22 (44.9%)	0.573
	F	3 (50.0%)	27 (55.1%)	
History of ameloblastoma	Yes	5 (83.3%)	22 (44.9%)	0.088
	No	1 (16.7%)	27 (55.1%)	
Site	Maxilla	4 (66.7%)	6 (12.2%)	0.013
	Mandible	2 (33.3%)	42 (85.7%)	
	Fronto-ethmoidal	0	1 (2.1%)	
Size	Mean ± SD	3.2 ± 1.8 cm	3.5 ± 1.6 cm	0.657
	(Range)	(1.2–6.0 cm)	(0.8–7.5 cm)	
	Median	3.0 cm	3.0 cm	
	(IQR)	(1.7–4.8 cm)	(2.5–4.0 cm)	
Histotype	Follicular	2 (33.3%)	28 (57.1%)	0.383
	Plexiform	1 (16.7%)	6 (12.2%)	
	Other	3 (50.0%)	15 (30.7%)	
Margins	Negative	5 (83.3%)	40 (81.6%)	0.47
	Positive	0	7 (14.3%)	
	Not known	1 (16.7%)	2 (4.1%)	
Reconstruction	Free flap	2 (33.3%)	23 (49.0%)	0.057
	Temporalis muscle flap	2 (33.3%)	3 (6.1%)	
	Bone graft	1 (16.7%)	9 (18.4%)	
	Plate	0	3 (6.1%)	
	No reconstruction	1 (16.7%)	10 (20.4%)	

**Table 6:** Univariate and multivariate logistic analyses.

Variables	Univariate Analysis		Multivariate Analysis	
	OR (95% CI)	*p*-Value	OR (95% CI)	*p*-Value
**Age**	2.4 (0.3–22.5)	0.436	0.8 (0.0–13.3)	0.876
**Anamnesis**	6.1 (0.7–56.5)	0.109	75.3 (0.8–6932.2)	0.061
**Site**	7.0 (1.6–29.6)	0.009	54.5 (1.7–1716.2)	0.023
**Size**	0.9 (0.5–1.7)	0.705	0.7 (0.3–1.8)	0.416
**Histotype**	0.7 (0.2–2.7)	0.592	2.4 (0.3–17.8)	0.382

## Data Availability

The data presented in this study are available on request from the corresponding author.
